# Psychophysiological Responses to a Brief Self-Compassion Exercise in Armed Forces Veterans

**DOI:** 10.3389/fpsyg.2021.780319

**Published:** 2022-01-18

**Authors:** Samantha Gerdes, Huw Williams, Anke Karl

**Affiliations:** ^1^Mood Disorder Centre, College of Life and Environmental Sciences, University of Exeter, Exeter, United Kingdom; ^2^The Veterans’ Mental Health and Wellbeing Service, Camden and Islington NHS Trust, London, United Kingdom

**Keywords:** self-compassion, veterans, PTSD, hyperarousal, loving-kindness, heart rate variability, skin conductance

## Abstract

Armed Forces personnel are exposed to traumatic experiences during their work; therefore, they are at risk of developing emotional difficulties such as post-traumatic stress disorder (PTSD), following traumatic experiences. Despite evidence to suggest that self-compassion is effective in reducing the symptoms of PTSD, and greater levels of self-compassion are associated with enhanced resilience, self-compassion in armed forces personnel and armed forces veterans remains under-researched. As a result, it is not known if therapeutic approaches that use self-compassion interventions are an acceptable and effective treatment for this population. Having previously shown that a one-off self-compassion exercise has temporary beneficial psychophysiological effects in non-clinical participants, we conducted this proof-of concept study to investigate whether this exercise is equally beneficial in veterans who had experienced deployment to a combat zone. Additionally, we examined if brief a self-compassion exercise can temporarily reduce hyperarousal symptoms and increase feelings of social connectedness. The current study also investigated the association between PTSD symptom severity, emotion regulation, and self-compassion in 56 veterans. All participants listened to a loving-kindness meditation for self-compassion (LKM-S) and psychophysiological recordings were taken throughout. Psychophysiological effects were observed including heart-rate (HR), skin conductance (SCL), and heart-rate variability (HRV) to determine associations with PTSD and changes in response associated with the self-compassion induction. PTSD symptom severity, dispositional emotion regulation, and self-compassion were measured, and participants also completed state measures of hyperarousal and social connectedness before and after the LKM-S. The findings partially demonstrated that self-compassion can be elicited in a veteran population but there were considerable individual differences in psychophysiological responses. The findings are discussed in light of existing theories of PTSD and self-compassion and the implications of using self-compassion based psychological approaches with veterans.

## Introduction

The impact of war-related trauma on soldiers is now well recognized and exposure to traumatic events while carrying out occupational duties can put armed forces personnel at an increased risk for developing PTSD (Dunn et al., 2015). Reports of PTSD prevalence rates in the armed forces population vary widely (Kok et al., 2012) with some estimating rates of PTSD in currently serving armed forces personnel at 4% (Hotopf et al., 2003; Fear et al., 2010); however, rates rise exponentially when soldiers are exposed to combat during deployments (11.9–22.5%; Kang et al., 2003) and PTSD rates increase with greater exposure to enemy contact and firefights (Hoge et al., 2004).

One particular characteristic of combat-related PTSD is a pattern of hypervigilance symptomology that is different from those found in civilian populations (Kimble et al., 2013). Individuals suffering from combat-related PTSD report enhanced physiological reactivity, an overactive startle response and emotional numbness compared with those who experience civilian traumatic events (Prescott, 2012). For soldiers in war zones, hypervigilance is highly adaptive such as the constant sensory scanning and searching (e.g., listening for footsteps and weapon sounds or looking for rising dust and shadows; (Department of the Army, 1984). Due to the constant threat to life or of serious physical injury endured for long periods of time, the hypervigilance is reinforced while on deployment (Kimble et al., 2013). As a result, it can become habitual and triggered easily, and difficult to eradicate once back in civilian life. Individuals are on constant “high alert” even when threat is low (Kimble et al., 2013), thus becoming problematic in civilian life as it can lead to disruptions in functioning such as increased aggression and sleep problems (Germain and Neilsen, 2003; Taft et al., 2007; Conoscenti et al., 2009).

Within the cognitive model of PTSD, elevated levels of hypervigilance and being in a threatened state maintains PTSD in combat veterans as it can prevent adaptive changes to the trauma memories (Ehlers and Clark, 2000). Veterans might hold a belief that hypervigilance was what enabled them to survive the traumatic experience and that they therefore cannot give it up. This could facilitate engagement in maladaptive coping strategies and safety behaviors such as constantly being on high alert and on the lookout for danger (Conoscenti et al., 2009). The drive to avoid feelings of threat further reinforces hypervigilant behaviors and prevents adaptive processing of the traumatic events. Individuals with combat-related PTSD, therefore, find it more difficult to engage in psychological therapies where exposure to traumatic memories is at the core (e.g., Ehlers and Clark, 2009).

Hypervigilance and other PTSD symptoms can leave people feeling detached and estranged from others and having difficulties experiencing positive feelings (American Psychiatric Association, 2013) as well as a feeling of difference, or “having changed” since the traumatic event (Demers, 2011). This can lead to difficulties in maintaining relationships (King et al., 2006) resulting in a lack of social support which can further contribute to a deterioration in mental health (Freedman et al., 2015). Further, the effect of the transition to civilian life is that the social support network experienced during a career with the armed forces is no longer available; hence, the combat veterans face an imminent lack of belongingness and social connection that may have been adaptive during their service (DeVries et al., 2003; Tick, 2005; Wessely, 2006). The profound beneficial effects of social support (Brewin et al., 2000; Ozer et al., 2003) and perceived social connectedness for recovery from psychological trauma and reducing PTSD have been demonstrated (Freedman et al., 2015). For example, post-deployment social support is negatively associated with PTSD in combat veterans (Pietrzak et al., 2009). However, the masculinized culture of the armed forces that promotes emotional stoicism (Reit, 2009; McAllister et al., 2018; Neilson et al., 2020) can prevent people from sharing emotional distress, and this may be further compounded during civilian life where veterans may feel alone in their experiences (Demers, 2011).

A lack of social support, difficulties in social relationships, or threats to social connection contribute to PTSD severity (Freedman et al., 2015) and also can activate the same stress response system as physical threats to survival, i.e., the fight/flight response, including the sympathetic nervous system (SNS) and the Hypothalamus-pituitary–adrenal (HPA) axis (Eisenberger and Cole, 2012). The combination of threats to social connection and hypervigilance due to fragmented, emotionally charged trauma memories can contribute to an elevated fight/flight response and hence mental health problems in veterans (Southwick et al., 2005). Elevated physiological arousal (Pole, 2007) and an elevated HPA response have been identified in people with PTSD. This elevated fight/flight state (i.e., hyperarousal) and constant activation of threat mode (i.e., hypervigilance), combined with a lack of social support may maintain PTSD in combat veterans, as individuals might be stuck in “current threat” mode (Ehlers and Clark, 2000).

Therefore, therapeutic approaches that emphasize reducing hyperarousal and the stress response as well as building social connectedness could reduce PTSD symptoms in veterans. The new concept of self-compassion has shown to be a promising approach for alleviation of PTSD symptoms in both civilian and veteran populations (e.g., Lee, 2009; Steen et al., 2021). Self-compassion can be described as “an intimate awareness of the suffering of oneself and others with the wish to alleviate it” (Germer and Neff, 2013). Dispositional self-compassion is negatively related to psychopathology (Barnard and Curry, 2011; MacBeth and Gumley, 2012) including PTSD and has been shown to predict recovery from PTSD (Thompson and Waltz, 2008; Meyer et al., 2019). Additionally, self-compassion can be cultivated in therapeutic settings and is therefore gaining popularity to treat a number of mental health difficulties (e.g., Germer and Neff, 2013, 2015) including PTSD and shame-based flashbacks (Lee, 2009; Daneshvar et al., 2020) as well as other shame-based difficulties (Leaviss and Uttley, 2015). Additionally, studies have shown that self-compassion could offer a protective process in preventing suicide in veterans, in times of distress as higher levels of self-compassion are associated with lower levels of psychopathology and suicidality (Kelley et al., 2019; Rabon et al., 2019).

Self-compassion could be beneficial for the treatment of PTSD in several ways: firstly, increasing self-compassion can reduce the “threat” emotion regulation system (Gilbert, 2009a) that is reflected by the excessive hyperarousal previously used as a survival mechanism and the negative self-appraisals that prevent adaptive processing and integration of the traumatic experience into the individual’s autobiographical memory (Ehlers and Clark, 2000). Both hyperarousal and negative self-appraisals are associated with activation of the sympathetic division of the autonomic nervous system as indicated by increased heart rate (HR) and skin conductance level (SCL; Pole, 2007) as part of the fight/flight response. In contrast, facilitating self-compassion secondly activates the “soothing and contentment” system (Gilbert, 2009a) characterized by a calm and content positive state and increased parasympathetic activation (as indicated by increased heart rate variability; HRV, Kirschner et al., 2019). This allows the individual not only to activate self-soothing and kindness but also to feel safe and socially connected (Gilbert, 2010). Increasing feelings of social connectedness *via* the activation of compassion represents the second possible mechanism *via* which it could reduce PTSD symptomology in veterans (e.g., Pearlman and Curtois, 2005; Freedman et al., 2015).

Facilitating self-compassion in a one-off meditation in civilian populations has been shown to increase perceived interpersonal connectedness (Hutcherson et al., 2008) and state secure attachment (Kirschner et al., 2019). However, the use of self-compassion with veterans with PTSD is in its infancy, though initial studies have found that self-compassion is negatively associated with PTSD (Dahm et al., 2015), that dispositional self-compassion levels are predictive of PTSD symptom severity (Hiraoka et al., 2015), and self-compassion is negatively related to maladaptive coping strategies such as impulsivity in military recruits (Mantzios, 2014). A 12-week course of loving kindness meditation (LKM) in veterans with PTSD led to an increase in self-compassion while symptoms of PTSD decreased (Kearney et al., 2013). Although self-compassion has demonstrated effectiveness for shame-based difficulties in PTSD (Lee, 2009), the mechanisms *via* which self-compassion interventions facilitate PTSD symptom reduction in veterans are not well understood to date.

It has not previously been studied whether facilitating self-compassion in veterans can reduce hypervigilance/hyperarousal as assessed by self-report and physiological measurements. In healthy individuals, a one-off short-term Loving Kindness Meditation for the Self (LKM-S) reduced physiological arousal symptoms; (i.e., reduced HR, SCL) and increased parasympathetic activation (i.e., increased HRV; Kirschner et al., 2019). In contrast, Creaser et al. (2021) found that civilian trauma survivors without PTSD and with subsyndromal and full PTSD who followed the same LKM-S had a reduction in negative self-perception and an increase in positive self-perception but did not show the expected physiological response pattern. Interestingly, individuals in the subsyndromal PTSD group who presented with higher levels of hyperarousal, showed a distinct physiological and brain response pattern, which indicated a threat response when instructed to direct compassion to the self. Similarly, in individuals with a history of recurrent depression, Kirschner et al. (2021) found that the LKM-S increased positive self-perception but this was not accompanied by the expected physiological response pattern. However, after completing an 8-week MBCT course, patients with recurrent depression showed a physiological pattern of calm and content positive affect (reduced HR and SCL and increased HRV; Kirschner et al., 2021).

In veterans, one prior study suggests that LKM can reduce PTSD symptoms (e.g., (Kearney et al., 2013) although to our knowledge there have been no further empirical studies to support this. The aim of the current study is to investigate the effects of a short-term one-off self-compassion meditation (LKM-S) in veterans who have experienced deployment to a combat zone. The pre-post changes on self-report hyperarousal symptoms (DSM-5, PTSD Cluster E) and feelings of social connectedness were examined. Additionally, we investigated physiological reactions during the self-compassion meditation to better understand the effects of the meditation on the fight/flight response. Specifically, we hypothesized that self-compassion would be cultivated in veterans, in both those who did and did not have PTSD as indicated by an increase on the self-compassion questions (Hypothesis 1). Additionally, we predicted that following the LKM-S there would be a decrease in self-reported hyperarousal symptoms (Hypothesis 2a) and a reduction in HR and SCL as measures of physiological arousal (Hypothesis 2b). We further predicted that there would be an increase in self-reported social connectedness (Hypothesis 3a) and an increase in HRV as a measure of parasympathetic activation (Hypothesis 3b). Given that more severe PTSD presentations can take longer to respond to psychological interventions (Hogberg et al., 2008), we also predicted that PTSD severity and emotion suppression would be negatively associated with the increase of LKM-S related self-compassion, social connectedness and HRV (Hypothesis 4a) and with the reduction in state hyperarousal and physiological arousal as indicated by HR and SCL (Hypothesis 4b). Finally, we predicted that dispositional self-compassion would result in a reverse association pattern and be positively associated with the increase of LKM-S related self-compassion, social connectedness and HRV (Hypothesis 5a) and with the reduction in state hyperarousal and physiological arousal (Hypothesis 5b).

## Materials and Methods

### Research Design

The study used a repeated measures design to test Hypotheses 1, 2a, and 3a, using outcome score at Time 1 (pre-LKM-S) and Time 2 (post-LKM-S) as dependent variables for self-report state hyperarousal, social connectedness and self-compassion. For SCL, HR, and HRV (Hypotheses 2b and 3b) the response in relation to the baseline (prior to the LKM-S) per minute over 11 min formed the repeated measures. In addition, a one-sample design was used to determine significant change from the baseline that was set to zero. To investigate the role of individual differences (Hypotheses 4 and 5), a correlational design was applied.

### Participants

Fifty-six armed forces veterans (52 males, 4 females) took part in the study. They were recruited between November 2017 and April 2018 through local veteran charities, the UK National Health Service (NHS) and online *via* social media platforms. Participants were eligible if they had experienced deployment to a combat zone during their career in the armed forces and they experienced combat or significant exposure to danger during the deployment. They were excluded from the study if they had a severe mental health problem such as schizophrenia or were acutely suicidal. Participants were also excluded if they had a prior history of cardiovascular problems including those that reported having had heart surgery, heart attacks or being on any cardiovascular medication. All participants gave written informed consent and the protocol was approved by the South West – Cornwall and Plymouth Research Ethics Committee (LREC) and the School of Psychology Ethics Committee of the University of Exeter.

Target sample size was determined *a priori* using a power calculation applying G*Power (Faul et al., 2009). Based on a medium effect size, it was calculated that 54 participants were needed to determine significant pre-to post changes in the dependent variables for a statistical power of 0.95 and alpha = 0.05. This sample size was deemed sufficient for examining the secondary correlational hypotheses with three predictors and an assumed large effect size (*f*^2^ = 0.35), however if only a medium effect size is obtained (e.g., *f*^2^ = 0.15) a larger sample size (*n* = 77), for a power of 0.80 and alpha = 0.05 would be necessary. We managed to recruit the target sample for Hypotheses 1–3.

The demographic and clinical information about the final sample can be seen in Table 1. The prevalence of PTSD was *n* = 19 in the current sample (those who had received a previous diagnosis from a psychiatrist). Based on scores on the PCL-5, *n* = 15 (26.8%) currently met criteria for PTSD, *n* = 5 (8.9%) met criteria for Subsyndromal PTSD^1^ on the PCL-5 and *n* = 36 (64.3%) did not have PTSD. All apart from one of the participants had PTSD symptoms as a result of their deployment experiences to a war zone, *n* = 18 (34%). One participant had PTSD as a result of an accident on a training operation during a non-combat deployment. All participants had been deployed to a combat zone which included conflicts such as the Falkland Islands, Northern Ireland, Kosovo, Iraq, and Afghanistan. Deployment length ranged from approximately two months to three years (including leave periods). There was an average of M = 4.06 (SD = 2.61; Median and Mode = 3) deployments to combat zones per participant.

**Table 1:** Demographic information for participants.

Characteristic	*N* = 56
**Gender, no. %**	
Female	4 (7.1)
Male	52 (92.9)
Age	52.1 (12.90)
**Marital Status, no. %**	
Married	38 (67.9)
Single	5 (8.9)
Divorced/separated	5 (8.9)
Cohabiting	4 (7.1)
Engaged	4 (7.1)
**Religion, no. %**	
No religion	22 (39.3)
Church of England	24 (42.9)
Catholic	3 (5.4)
Buddhist	2 (3.6)
Methodist	1 (1.8)
Other	2 (3.6)
Not stated	2 (3.6)
**Occupation, no. %**	
Employed FT	27 (48.2)
Employed PT	13 (23.2)
Retired	15 (26.8)
Unemployed	1 (1.8)
**Nationality, no. %**	
British	54 (96.4%)
Dual British Nationality	2 (3.6%)
**Armed Forces Branch, no. %**	
Army	13 (23.2%)
Royal Navy	6 (10.7%)
Royal Marines	28 (50.0%)
Royal Air Force	6 (11.0%)
Army Reserves	1 (1.8%)
Royal Marines Reserves	1 (1.8%)
Special Forces	1 (1.8%)
**Rank at discharge, no. %**	
Colonel	1 (1.8)
Lieutenant-Colonel	4 (7.1)
Major/Lieutenant Commander	6 (10.7)
Captain/Flight Lieutenant	7 (12.5)
Sub-Lieutenant	1 (1.8)
Sergeant Major	1 (1.8)
Warrant Officer 1^st^ Class	4 (7.1)
Warrant Officer 2^nd^ Class	1 (1.8)
Sergeant	9 (16.1)
Corporal/Leading Hand	9 (16.1)
Lance Corporal/Junior technician	5 (8.9)
Private/Marine/Senior Aircraftman	7 (12.5)
**Physical injury on deployment, no. %**	
Yes	27 (48.2)
No	29 (51.8)
**PTSD from combat experiences, no. %**	
Yes	18 (32.1)
No	38 (67.9)

There were *n* = 27 (48.2%) participants who had sustained a physical injury while on deployment. Forty participants (71.4%) had experienced at least one Traumatic Brain Injury (TBI) as classified by the work of Williams et al. (2010) (see Table 2).

**Table 2:** Traumatic Brain Injury Assessment (Williams et al., 2010).

Classification	*N* = 56 (%)
0 = No history	15 (26.8)
1 = Feeling dazed and confused but no LOC, minor concussion	1 (1.8)
2 = LOC < 10 min, mild TBI	24 (42.9)
2a = LOC but no concussion symptoms	14 (25.0)
3 = LOC 10 to 30 min, complicated mild TBI	1 (1.8)
4 = LOC 30 to 60 min, moderate/severe TBI	1 (1.8)
5 = LOC > 60 min, very severe TBI	0

LOC = Loss of consciousness. TBI was assessed over the participant’s lifetime rather than restricted to just their armed forces career.

### Measures and Materials

#### Self-Report Measures

The PTSD Checklist for DSM-5 (PCL-5; Weathers et al., 2013) consists of 20 items, rated on a five-point Likert-scale, assessing the twenty DSM-5 symptoms of Post-Traumatic Stress Disorder. Validation studies for the PCL-5 show strong internal consistency (*a* = 0.94), test–retest reliability (*r* = 0.82), and convergent (*r*s = 0.74–0.85) and discriminant validity (*r*s = 0.31–0.60; Blevins et al., 2015).

The Patient Health Questionnaire for depression (PHQ-9; Kroenke et al., 2001) consists of 9 items, rated on a four-point Likert-scale, which is used to establish levels of depression in primary care and other medical settings. The PHQ-9 has excellent reliability, internal = 0.89 and test re-test = 0.84 validity for detecting depression = 0.95 (Solomon et al., 2000).

The Emotion Regulation Questionnaire (ERQ, Gross and John, 2003) consists of 10 items, rated on a seven-point Likert-scale, assessing the ability to regulate emotions in terms of cognitive reappraisal^2^ and expressive suppression.^3^ Prior research has shown that the ERQ has high internal reliability, and convergent and discriminant validity (Gross and John, 2003).

The Self-Compassion Scale-Short Form (SCS-SF; Raes et al., 2011), consists of 12 items rated on a five-point Likert-scale assessing trait level of self-compassion. The short form is near perfectly correlated with the Self Compassion Scale (SCS) *r* = 0.98 (Raes et al., 2011) and the scale has demonstrated validity and reliability (Neff, 2003a).

Visual Analogue scales (ranging from 0 to 100) were used to establish state levels of self-compassion, hyperarousal and social connectedness before and after the LKM-S. State self-compassion was used as a manipulation check to determine self-compassion induction in participants and also engagement with the meditation. The VAS measure is adapted from Kirschner et al. (2019) and questions are taken from the Self-Compassion Scale (SCS; Neff, 2003a), social connectedness questions are based on the state adult attachment measure (SAAM; Gillath et al., 2009) and four adapted items from the PCL-5 have been added to measure state hyperarousal. The VAS has been used in previous studies (Kirschner et al., 2019, 2021) which found Cronbach’s *α* = 0.66 for state affiliative affect, state self-compassion (Cronbach’s *α* = 0.73 in this sample) and state self-criticism (Cronbach’s alpha in this sample was 0.73 for the inadequate self, 0.76 for the hated self, and 0.77 for reassure self).

#### Loving Kindness Meditation

A self-compassion meditation (LKM-S) was used to induce self-compassion in the current study. The LKM-S has been developed by the ACCEPT clinic, at the University of Exeter Mood Disorder Centre. The LKM-S audio clip was recorded by an experienced mindfulness practitioner, and the LKM-S has been used in prior research (e.g., Kirschner et al., 2019). Participants are asked to direct loving/friendly feelings toward themselves and others and the audio clip is 11.5 min in length.

### Physiological Measurements

All physiological parameters were recorded continuously using a BIOPAC MP150 system using the AcqKnowledge 4.2 (BIOPAC Systems; Goleta, CA) software. HR and HRV was determined from the electrocardiogram (ECG) using standard procedures (Berntson et al., 1997; Berntson and Stowell, 1998). ECG was recorded from below the participant’s right collar bone and the participant’s left lower ribcage using a BIOPAC ECG100C amplifier at a sampling rate of 1 kHz with a low pass filter of 0.5 Hz and a high pass filter of 35 Hz. Skin conductance levels (SCL) were recorded using a BIOPAC SCL100C amplifier and a skin resistant transducer (TSD203) from the middle phalanx of the first and second fingers of the participant’s non-dominant hand, at a sampling rate of 500 Hz with a low pass filter of 1.0 Hz. SCL was pre-processed using recommended procedures (Lykken et al., 1966).

### Procedure

Participants were self-selected and were recruited from a range of veteran organizations and charities in the South West of England, NHS services in Devon and online social media adverts. Ninety-two people expressed an interest in the study and 81 people completed the telephone screening. Seventy participants were eligible and signed up to take part in the study, 14 participants dropped out at this stage due to reasons such as work commitments and illness. A total of 56 participants completed the study. Eligible participants were booked in for a testing session at the University of Exeter following a telephone screening call. The study procedure, which lasted approximately 1–1.5 h, included collecting demographic information, completing psychometric measures, and listening to the compassion meditation (LKM-S) while physiological recordings were taken. Standardized instructions were given for the VAS questions and LKM-S audio. The participants listened to the LKM-S in a quiet room in the psychophysiological laboratory at the University of Exeter. Instructions were given to the participants and they were given the opportunity to ask questions, before the researcher left the room and the participant listened to the LKM-S audio. All participants received a reimbursement for their time of £10.

### Data Analysis

There was missing psychophysiological data for one participant therefore the analysis for physiological data is based on 55 participants. No other missing data were detected in the data set. Outliers were detected after examining boxplots, however inspection of their values did not reveal them to be extreme, so they were kept in the analysis in order to include all participants in the analysis. The outlying data points were changed to the next closest value that was under the cut off, which is a technique for dealing with outliers, while maintaining the shape of the sample distribution but the outliers do not distort the data (Tabachnick and Fidell, 2007). Assumptions of normality were not violated. For the regression analysis, assumptions of independence of observations, linearity, homoscedasticity, normality, and multicollinearity were all fulfilled.

Data pre-processing and further statistical analyses of the psychophysiological data followed established procedures; i.e., determining the size of the response in relation to a pre-induction baseline as per previous studies (Kirschner et al., 2019). To test Hypotheses 1a, 2a, and 3a, paired sample *t*-tests were used to examine pre and post scores. Repeated measures ANOVAs over the 11 timepoints were conducted to investigate if there is a significant main effect of time. One sample *t*-tests were used for the psychophysiological data to determine whether scores differed from zero (i.e., HRV; as index of parasympathetic activation and HR, SCL as measure of sympathetic arousal; Hypotheses 2b and 3b). Correlations and regression analysis were used to examine the associations between responses to the LKM-S and individual differences in PTSD severity, emotion regulation, and dispositional self-compassion (Hypotheses 4 and 5). For this, residualized gains scores (RGS; Mintz et al., 1979; Williams et al., 1984) were calculated for state hyperarousal, self-compassion, and social connectedness, which were used as outcome variables.

## Results

### Change in State Self-Compassion (Hypothesis 1)

**Figure 1:**
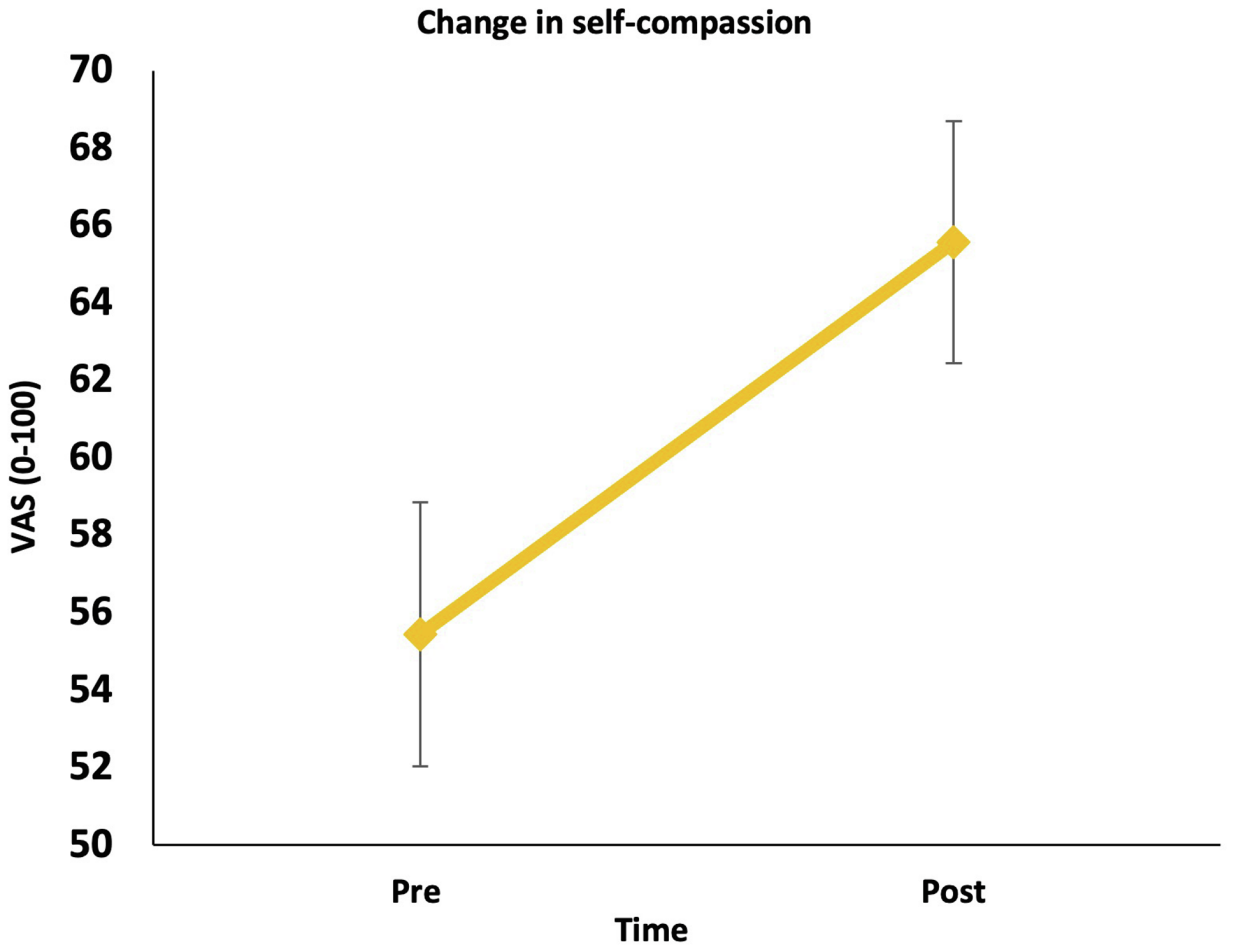
Change in state self-compassion (pre to post).

In line with Hypothesis 1, there was a significant increase in state self-compassion from pre to post LKM-S, *F* (1, 55) = 13.62, *p* = 0.001, *ηp*^2^ = 0.199 (Figure 1).

### Change in State Hyperarousal (Hypothesis 2a)

**Figure 2:**
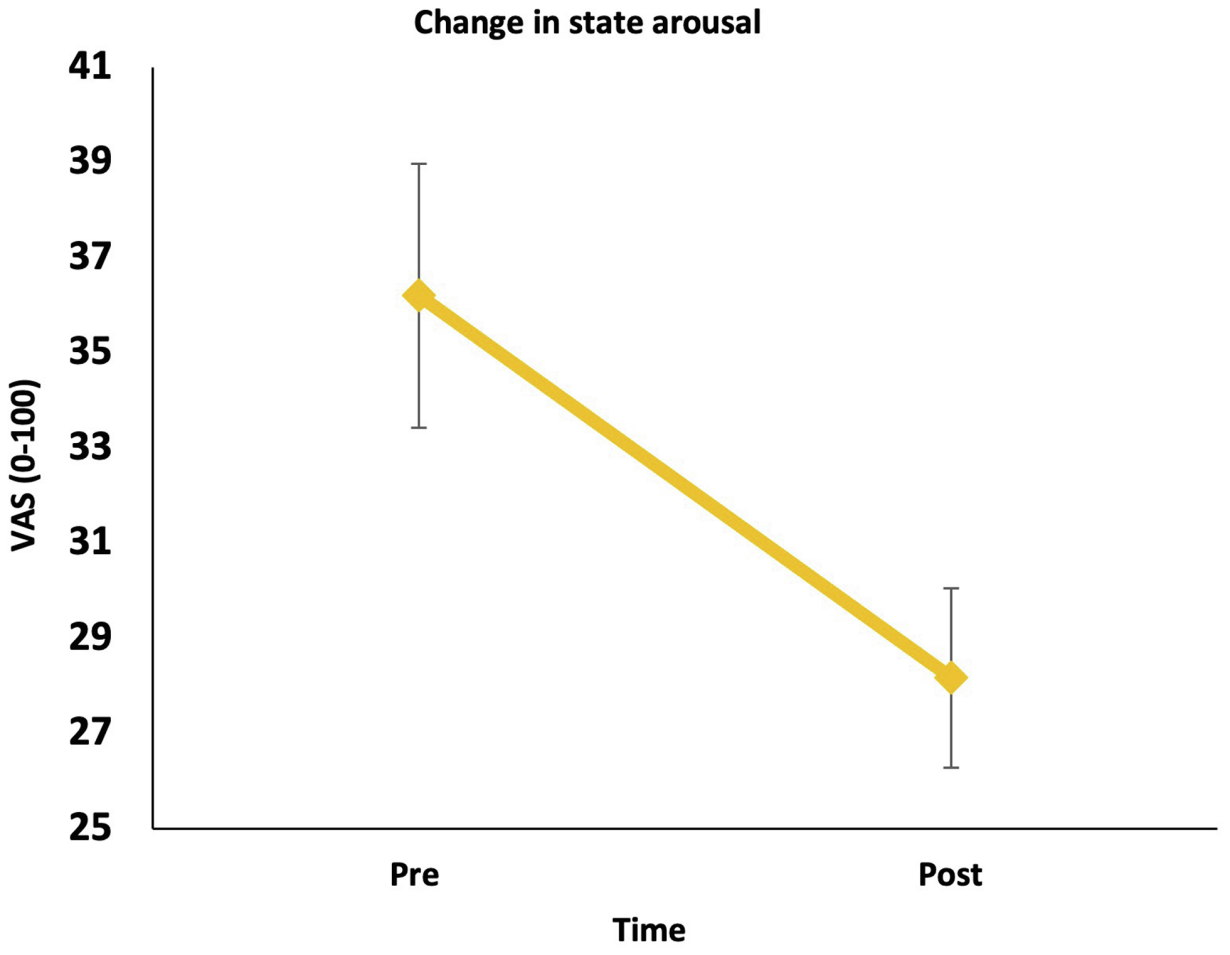
Change in state hyperarousal (pre to post).

In line with our hypothesis, there was a significant reduction in state arousal pre to post the LKM-S, *F* (1, 55) = 17.59, *p* < 0.001, *ηp*^2^ = 0.242 (Figure 2).

### Physiological Arousal Response (Hypothesis 2b)

#### Heart Rate Response

**Figure 3:**
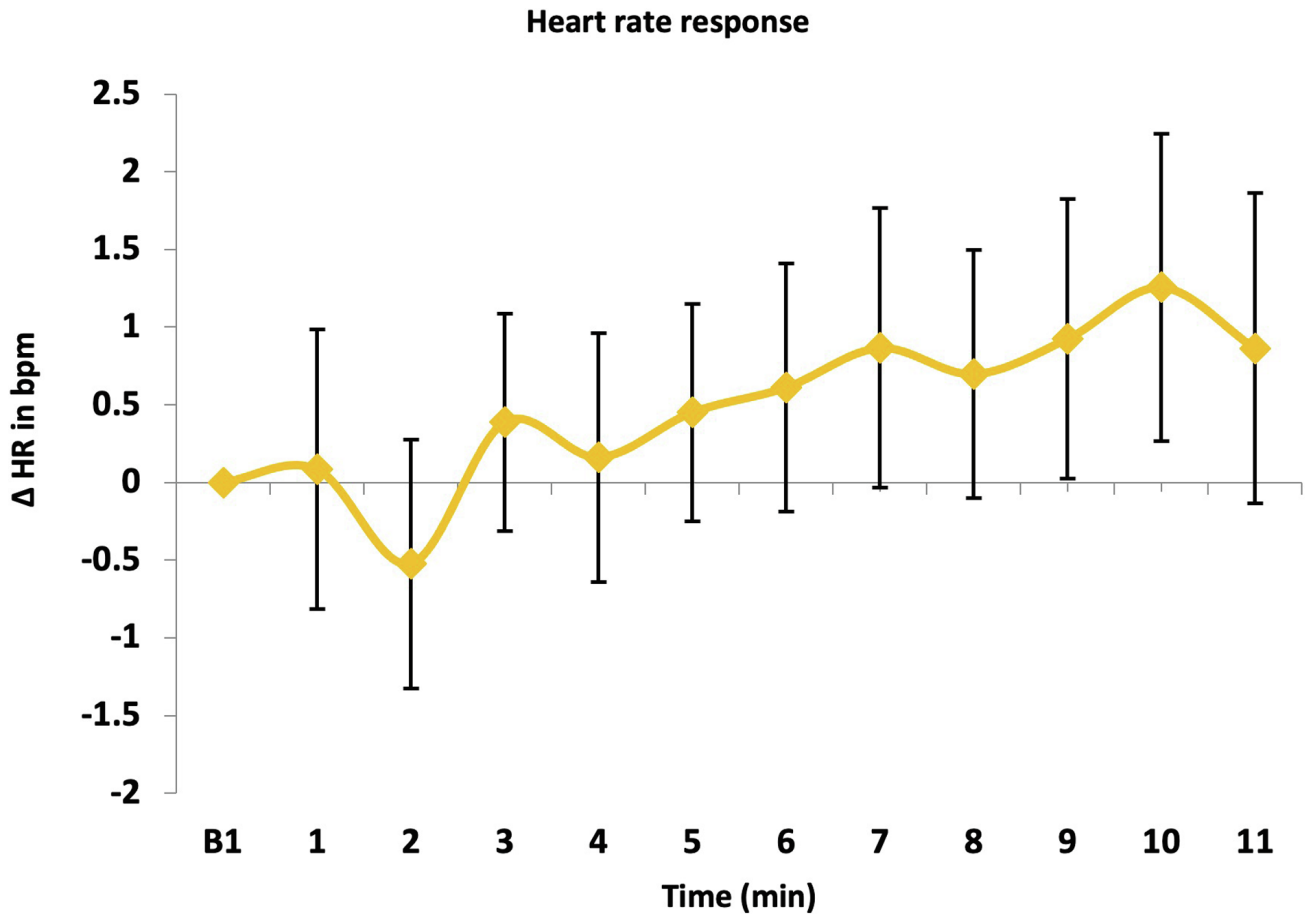
Heart rate (HR) response over time.

Overall, there was a significant effect of time on HR response, *F* (10, 45) = 3.57, *p* = 0.002, ηp^2^ = 0.442 (Figure 3) suggesting a rise of HR toward the end of the LKM-S. Although the one-sample t-test revealed that mean HR response (M = 0.52, SD = 2.71) did not significantly differ from zero, *t*(54) = 1.68, *p* = 0.09, Cohen’s *d* = 0.27, it is of interest that, the one-sample t-test revealed that HR response to directing compassion towards the self (6–11 min; M = 0.87, SD = 3.36) revealed a significantly increased HR, *t*(54) = 2.01, *p* = 0.04, Cohen’s *d* = 0.27. This indicates that a one-off LKM-S did not significantly reduce physiological arousal as indicated by HR, on the contrary, there is indication of an increase in physiological arousal when individuals direct compassion toward themselves.

#### Skin Conductance Level Response

**Figure 4:**
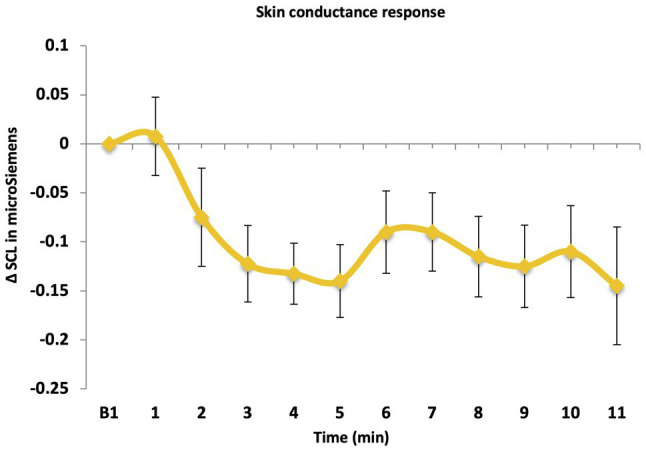
Skin conductance (SCL) response over time.

Overall, there was a significant effect of time, *F*(10, 45) = 6.00, *p* < 0.001, *ηp*^2^ = 0.571 (Figure 4) suggesting a reduction of SCL. A one-sample *t*-test revealed that the mean SCL response (*M* = −0.04, *SD* = 0.07) was significantly lower than zero, *t*(54) = −4.33, *p* < 0.001, Cohen’s *d* = −0.58. This indicates that there was a reduction in sympathetic arousal as indicated by SCL, during the one-off LKM-S, and a medium effect size was observed.

### Change in Social Connectedness (Hypothesis 3a)

**Figure 5:**
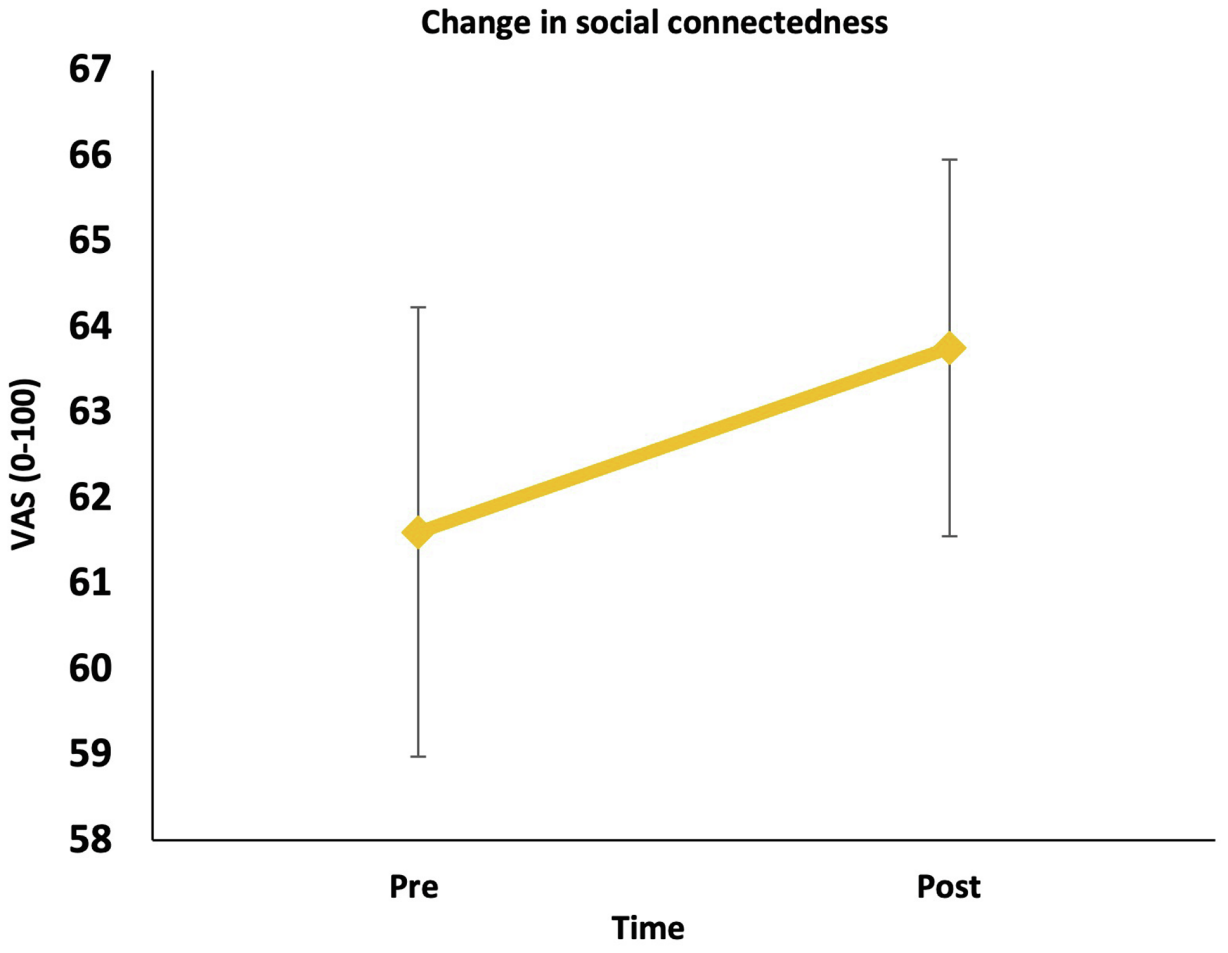
Change in social connectedness (pre to post).

Contrary to our hypothesis, there was no significant increase in social connectedness from pre to post the LKM-S, *F* (1, 55) = 2.36, *p* = 0.130, *ηp*^2^ = 0.041 (Figure 5).

### Parasympathetic Response (Hypothesis 3b)

#### Heart Rate Variability Response

**Figure 6:**
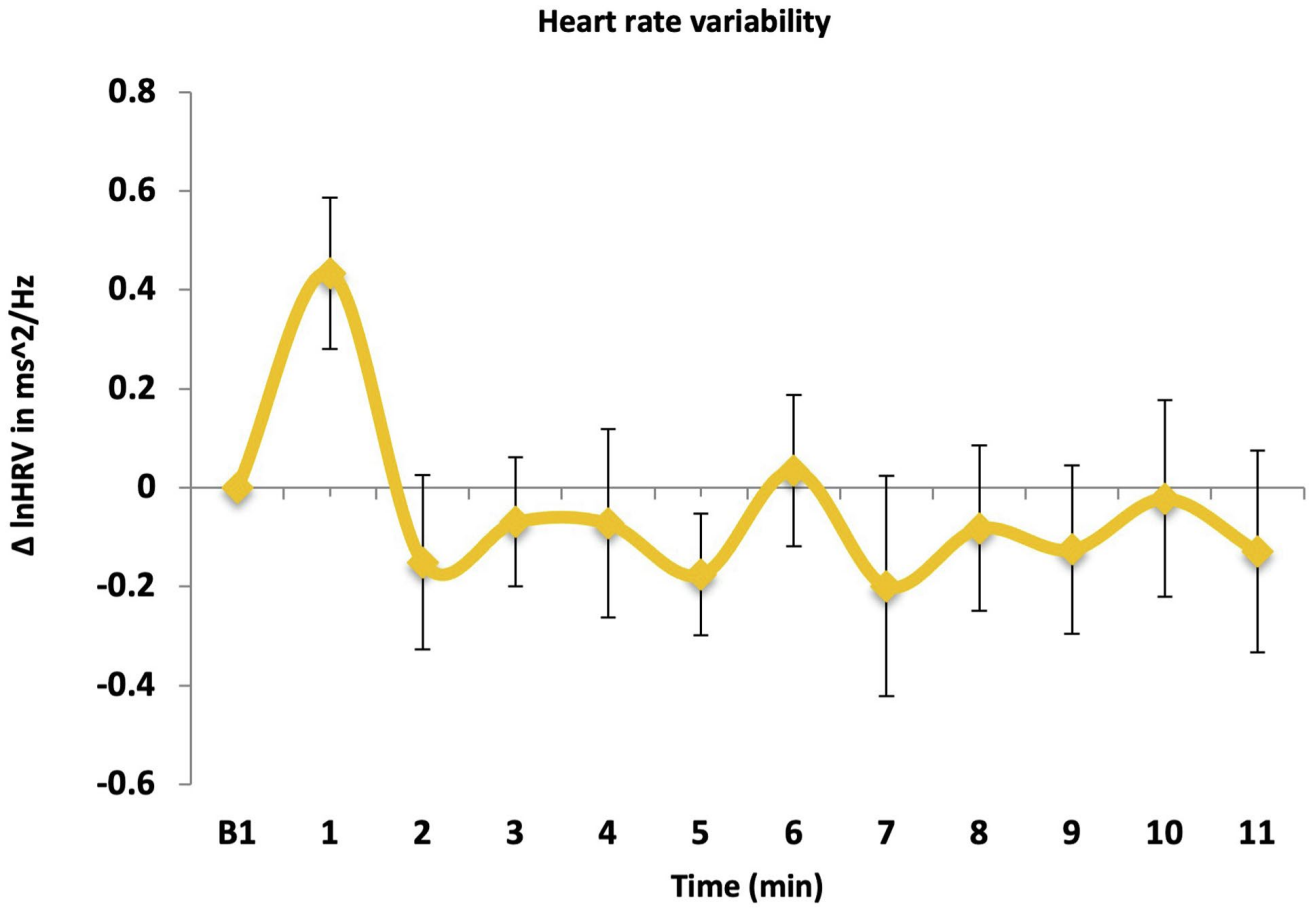
HR variability over time.

There was a significant effect of time, *F* (10, 45) = 2.57, *p* = 0.015, *ηp*^2^ = 0.364 (Figure 6). A one-sample *t*-test revealed that mean HRV response (M = −0.05, *SD* = 1.06) did not significantly differ from zero, *t*(54) = −0.61, *p* = 0.54, Cohen’s *d* = −0.08. This indicates that a one-off LKM-S did not significantly increase parasympathetic activation as indicated by HRV.

### Role of Individual Differences (Hypotheses 4 and 5)

#### Effects of Individual Differences on Change in Self-Compassion

A multiple regression was used to predict the change in self-compassion, as measured by the VAS, pre–post the loving kindness meditation (LKM-S). Overall, the regression model was significant, *F*(1,53) = 4.08, *p* = 0.048; *R*^2^ = 0.07, and explained 7% of variance. Overall, levels of state self-compassion at both pre and post time points were associated with PTSD (*r* = −0.496, *p* < 0.001), trait self-compassion (*r* = 0.498, *p* < 0.001) and emotion suppression (*r* = −0.296, *p* = 0.027). However, PTSD severity, trait self-compassion, and emotion suppression were not significantly associated with LKM-S-induced state self-compassion change. Instead, the greater increase in state self-compassion was associated with reduced skin conductance to compassion for others (first 5 min of the LKM-S), *β* = −0.267, *t* = −2.02, *p* = 0.048.

#### Effects of Individual Differences on Change in Social Connectedness

A multiple regression was used to predict the change in social connectedness pre to post the LKM-S. Overall, the regression model was significant, *F*(1,53) = 4.26, *p* = 0.044; *R*^2^ = 0.07, and explained 7% of variance. Though the levels of social connectedness at both time points were negatively associated with PTSD (*r* = −0.649, *p* < 0.001), self-compassion (*r* = 0.594, *p* < 0.001) and emotion suppression (*r* = −0.433, *p* = 0.001), PTSD severity, trait self-compassion, and emotion suppression were not significantly associated with LKM-S-induced change in social connectedness. Instead, greater increase in social connectedness associated with higher baseline HRV, *β* = 0.273, *t* = 2.06, *p* = 0.044.

#### Effects of Individual Differences on Change in State-Hyperarousal

A multiple regression was used to predict the change in state-hyperarousal, as measured by the VAS, pre–post the loving kindness meditation (LKM-S). Overall the regression model was significant, *F*(2,52) = 9.22, *p* < 0.001; *R*^2^ = 0.26, and explained 26% of variance. Although state arousal at both time points was associated with PTSD (*r* = 0.768, *p* < 0.001), self-compassion (*r* = −0.453, *p* < 0.001) and emotion suppression (*r* = 0.318, *p* = 0.017), PTSD severity, trait self-compassion and emotion suppression were not significantly associated with LKM-S-induced arousal change. Instead, greater state arousal reduction was associated with state change in social connectedness, *β* = −0.403, *t* = −3.37, *p* = 0.001, and baseline heart rate, *β* = 0.347, *t* = 2.90, *p* = 0.005.

#### Effects of Individual Differences on Heart Rate Response

A regression model was used to predict HR response in compassion to others, which was significant: *F*(1,53) = 4.51, *p* = 0.038; *R*^2^ = 0.08 (8% of variance explained). However, only emotion suppression was significantly associated with change in HR when directing compassion to others, *β* = 0.280, *t* = 2.12, *p* = 0.038.

#### Effects of Individual Differences on Skin Conductance Level Response

PTSD severity, trait self-compassion, and emotion suppression were not significantly associated with LKM-S-induced SCL response.

#### Effects of Individual Differences on Heart Rate Variability Response

A regression model was used to predict the HRV response when directing compassion to the self, which was significant, *F*(1,53) = 5.54, *p* = 0.022; *R*^2^ = 0.10, and explained 10% of variance. However only trait self-compassion was significantly associated with change in HRV when directing compassion to self, *β* = 0.308, *t* = 2.35, *p* = 0.022.

### Exploratory Analyses: Traumatic Brain Injury

No significant associations were found between TBI severity and any of the variables.

## Discussion

This study aimed to investigate the effects of a brief, one-off loving kindness meditation for the self (LKM-S) on state self-compassion, hyperarousal, social connectedness, and physiological responses, in armed forces veterans who had experienced deployment to a combat zone. Following one’s listening to the LKM-S, participants had a significant increase in state self-compassion and a significant reduction in self-reported hyperarousal. Additionally, there was a decrease in the SCL response following the LKM-S. However, there was no increase in state social connectedness and there was not the expected increase in HRV and decrease in HR. Interestingly, the physiological responses were partially associated with individual differences in trait self-compassion and emotion regulation but not with PTSD severity as we predicted.

Overall, the findings are partially in line with previous research by Kearney et al. (2013) who found that veterans who took part in a 1.5 h weekly loving-kindness meditation course over 12 weeks, had increased levels of state self-compassion and reduced PTSD symptoms, including levels of state arousal, immediately after the treatment and at a 3-month follow-up. Additionally, changes in self-compassion mediated the changes in PTSD symptoms pre to post treatment. However, in the current study the changes in the physiological responses were not in line with our predictions except for SCL. We had hypothesized this in line with previous findings from Kirschner et al. (2019) who found that the LKM-S induced a pattern of reduced physiological arousal (HR and SCL reductions) and increased parasympathetic activity (HRV increase).

There are several possible explanations for the physiological findings of this study. First, there could be a “dose–response” effect, whereby a one-off administration of a short (11.5 min) LKM-S is not enough to have an impact at a physiological level in a clinical sample. In line with PTSD theories (Ehlers and Clark, 2000) and the tripartite model of emotion regulation (Gilbert, 2009a), it may be more challenging for trauma survivors, in particular those with higher PTSD symptoms, to switch from “threat and self-protection” system to the “soothing and contentment” emotion system. In contrast to the findings of the current study, a longer loving-kindness meditation course (Kearney et al., 2013) where veterans attended weekly sessions over 12-weeks, led to significant reductions in PTSD symptoms and also an increase in self-compassion. Additionally, the course was run by experienced mindfulness teachers who guided participants through loving-kindness meditations and also encouraged discussion around integrating loving-kindness meditation into everyday life. Participants were also provided with a book and CD to encourage practice between the weekly sessions. Comparing our and Kearney’s findings suggests that a longer and more in-depth loving-kindness meditation practice may be needed to establish changes on a physiological level in individuals with an overactivated threat system such as those with PTSD.

Second, and in support of the notion that an overactivated threat system may affect a person’s ability to engage effectively with a one-off LKM-S, the absence of the expected physiological effect is more in line with previous research in a sample of individuals with a history of recurrent depression, some of which had reported early childhood adversity (Kirschner et al., 2021). Prior to an 8-week course of mindfulness-based cognitive therapy (MBCT), the participants did not have a reduction in physiological arousal and an increase in parasympathetic activation after listening to the LKM-S, despite self-reported increases in state self-compassion, which is similar to our findings in a veteran population. On the contrary, participants in the current study showed an increase in physiological arousal through an elevated heart-rate, and in Kirschner et al. (2021) showed an indication of increased arousal and reduced HRV, when directing compassion to themselves, thus supporting the theoretical accounts cited above (Ehlers and Clark, 2000; Gilbert, 2009a). Interestingly, the group of recurrent depressed individuals in Kirschner et al. (2021) who completed the MBCT, which has been shown to increase dispositional self-compassion despite not having a direct compassion component (Kuyken et al., 2010), showed the predicted reduction in HR and SCL and increase in HRV post intervention, whereas the untreated control group showed a more elevated SCL response at the second exposure to the LKM-S. Taken together, our and the previous studies (Kirschner, 2021) suggest that a “one-off” self-compassion meditation in individuals with an overactivated threat system may result in a subjective effect for participants as recorded by self-report measures, but does not on an automatic, habitual level, which would have been reflected in participants’ physiological responses.

In contrast to our hypothesis, we also did not find that the one-off LKM-S increased a feeling of social connectedness as had been previously reported by Hutcherson et al. (2008) in civilian populations. Given that self-compassion approaches activate the “soothing and contentment” system, which is underpinned by the parasympathetic nervous system and enables feelings of social safety and connectedness (e.g., Gilbert, 2010), we hypothesized that participants would experience an increase in feelings of social connectedness after listening to the LKM-S. It might be that similarly to physiological responses, there is a “dose–response” effect, and participants need longer than a short meditation to experience changes in their felt experience of social connectedness. In addition, this may be due to an inherent lack of social support given that veterans have often needed to adapt to a different social system in civilian life after leaving the armed forces. This can mean that veterans are without the social networks and social support that were so adaptive during service in the armed forces (Wessely, 2006) and therefore might explain why we did not see the expected changes following the LKM-S in the current study.

We hypothesized that PTSD severity and emotion suppression would be negatively associated with the increase of LKM-S related self-compassion, social connectedness and HRV (Hypothesis 4a), given that it is well-established that severity of PTSD affects participants response to and ability to engage in psychological interventions (Hogberg et al., 2008). However, we did not find support for a role of PTSD symptoms. This was unexpected but could be due to the sample largely consisting of participants not fulfilling criteria for full or subsyndromal PTSD resulting in overall low levels of PTSD severity in our participants. Instead, we found that there was partial support for role of dispositional self-compassion and emotion suppression. Those with lower levels of self-compassion and those who use strategies such as emotion suppression, found it more difficult to engage with the LKM-S exercise, which is in line with the work by Gilbert (2010) around fears and blocks to compassion in those with low levels of dispositional self-compassion, i.e., people who have low levels of self-compassion can find it threatening to engage in compassion which is seen in an elevated threat response (Gilbert, 2010). Often, this is due to having experienced past trauma and especially those who have experienced interpersonal trauma, such as that in combat, are particularly susceptible to this aversive response. In addition, people who use emotion suppression as a strategy to manage emotions, find it more difficult to engage with and experience positive emotional states when they arise, such as social safety and connectedness associated with the “soothing and contentment” system (Gilbert, 2010). Although suppressing emotions can be adaptive for soldiers in combat situations and is likely to be reinforced as a habitual response (Neilson et al., 2020), it can cause problems once veterans are re-immersed in civilian life as it is difficult to establish social safety and feel connected to others (e.g., Prescott, 2012). Similarly to altering physiological responses, it is more difficult to change emotion suppression in veterans when it has been conditioned as part of their occupational role combat trauma (Prescott, 2012). Given the high percentage of mild traumatic brain injury (mTBI) in our sample, which could affect emotion regulation abilities and contribute to posttraumatic stress symptoms (Belanger et al., 2009; Mounce et al., 2013), we additionally explored the associations of mTBIs and response to LKM-S and our symptom and dispositional measures. No significant associations were revealed, which suggests that mTBIs were not associated with individuals’ ability to engage in a one-off self-compassion meditation.

## Limitations

Overall, the interpretation of the findings of the current study needs to take into account several limitations.

Firstly, there was not a control group of participants who were not exposed to the LKM-S. This means we were not able to determine whether the changes noted were due to other factors, such as becoming more comfortable in the surroundings. It would have been interesting to determine whether there were baseline differences in physiological responses in veterans who have experienced deployment to a combat zone vs. a civilian group of trauma survivors who have not held a role in the armed forces, as studies have shown that combat trauma results in higher levels of hyperarousal than does other types of trauma (Prescott, 2012). Additionally, there was no follow up after the study, so we are not aware for how long the changes in self-compassion were maintained. The VAS scale was used in the study as a self-report measure, and it is not known whether individual differences in emotional expression and motor control affected the responses across participants. In terms of the sample, there was an unequal proportion of men and women in the study, which is a common recruitment bias in veteran populations and is representative of the armed forces population. There was also a large difference in the severity of PTSD in participants – some had no symptoms at all, whereas others had very severe levels of PTSD. Additionally, the measurement of PTSD in the current study relied on self-report measures rather than clinician-administered interviews, so it might be that some participants felt unable to disclose their distress in the study setting. Another limitation of the sample is that although the screening process excluded participants who had not experienced combat or exposure to life threatening danger during their deployment to combat zones, the length of and number of deployments was not controlled for. Lastly, although the target sample size for the current study was calculated *a priori*, the sample size recruited meant that for the regression analyses, we were only able to detect medium-to-large effect sizes.

## Conclusion

Overall, the results demonstrate that there are temporary benefits of a one-off compassion meditation on self-report measures of self-compassion and hyperarousal but not on physiological responses. Our results suggest that a self-compassion-based approach appeared acceptable for veterans who are experiencing emotional distress including PTSD. Ours and previous findings considered together (Kearney et al., 2013; Kirschner et al., 2021) suggest that compassion-based approaches are potentially beneficial for clinical samples and could extend or complement existing psychological treatments for veterans with PTSD, given the well-established role of social support for recovery from trauma and PTSD, (Brewin et al., 2000; Ozer et al., 2003). The findings also highlight the importance of individual differences and the need for a longer-term intervention in such populations. Given the need for treatments for PTSD to create lasting changes that are on a psychological as well as physiological level, increasing HRV could be an important treatment target for development of better self-soothing/emotion regulation in trauma survivors (e.g., Arch et al., 2013). Additionally, reducing hyperarousal and increasing social connectedness are areas that can be targeted in treatment for PTSD.

## Future Research

Future studies should investigate the individual difference factors identified in the current study, such as the effect on the participant’s ability to engage with self-compassion approaches, including cultivating feelings of compassion toward oneself and others (e.g., Gilbert, 2010). For example, Creaser et al. (2021) found that people who had high levels of PTSD hyperarousal symptoms (also common in veterans) had a large threat response as indicated by physiological measures, when instructed to direct compassion toward oneself. Gaining further understanding of the effects of individual differences on ability to engage with self-compassion, could be key in understanding why psychological therapies for combat trauma are less effective compared with other types of trauma (Bradley et al., 2005); therefore, it is important that researchers continue to develop an understanding in this area. Additionally, it might be that there are different PTSD symptom patterns and a link between numbing/emotion suppression and threat response indicators, such as HR (Gutner et al., 2010), which also warrant further investigation. Activating self-compassion in veterans could provide a helpful avenue to address the issues of shame and moral injury (Litz et al., 2009; Saraiya and Lopex-Castor, 2016) that also appear to maintain hyperarousal symptoms in PTSD (Feiring and Taska, 2005) and can lead to social withdrawal and lack of social connectedness (Litz et al., 2009).

## Data Availability Statement

The raw data cannot be shared because the participants did not agree that their data can be shared at the consent stage. Requests to access the datasets should be directed to Anke Karl at A.Karl@exeter.ac.uk.

## Ethics Statement

The studies involving human participants were reviewed and approved by the South West – Cornwall and Plymouth Research Ethics Committee (LREC) and the School of Psychology Ethics Committee of the University of Exeter. The patients/participants provided their written informed consent to participate in this study.

## Author Contributions

SG, HW, and AK contributed to the conception and design of the study. SG and AK organized the database and performed the statistical analysis. SG wrote the first draft of the manuscript. AK wrote sections of the manuscript. All authors contributed to the article and approved the submitted version.

## Conflict of Interest

The authors declare that the research was conducted in the absence of any commercial or financial relationships that could be construed as a potential conflict of interest.

## Publisher’s Note

All claims expressed in this article are solely those of the authors and do not necessarily represent those of their affiliated organizations, or those of the publisher, the editors and the reviewers. Any product that may be evaluated in this article, or claim that may be made by its manufacturer, is not guaranteed or endorsed by the publisher.

## Footnotes

^1^Subsyndromal PTSD is categorized as endorsing one Cluster B symptom (intrusion symptoms), and one of either cluster C (avoidance), D (negative alterations in cognitions), or E (hyperarousal).

^2^Cognitive Reappraisal is cognitive change which can alter how we interpret a situation and therefore changes the emotional response (Lazarus and Alfert, 1964).

^3^Expressive Suppression is the ability to inhibit the emotive-expressive behavior triggered by an emotional response (Gross, 1998).

## References

[ref1] American Psychiatric Association (2013). Diagnostic and Statistical Manual of Mental Disorders. 5th Edn. Washington, DC: American Psychiatric Association.

[ref2] Arch J. J. Ayers C. R. Baker A. Almklov E. Dean D. J. Craske M. G. (2013). Randomized clinical trial of adapted mindfulness-based stress reduction versus group cognitive behavioural therapy for heterogeneous anxiety disorders. Behav. Res. Ther. 51, 185–196. doi: 10.1016/j.brat.2013.01.003, PMID: 23419887

[ref3] Barnard L. K. Curry J. F. (2011). Self-compassion: conceptualisation, correlates, and interventions. Rev. Gen. Psychol. 15, 289–303. doi: 10.1037/a0025754

[ref4] Belanger H. G. Kretzmer T. Vanderploeg R. D. French L. M. (2009). Symptom complaints following combat-related traumatic brain injury: relationship to traumatic brain injury severity and posttraumatic stress disorder. J. Int. Neuropsychol. Soc. 16, 194–199. doi: 10.1017/S1355617709990841, PMID: 19758488

[ref5] Berntson G. G. Bigger J. T. Eckberg D. L. Grossman P. Kaufmann P. G. Malik M.. (1997). Heart rate variability: origins, methods, and interpretive caveats. Psychophysiology 34, 623–648. doi: 10.1111/j.1469-8986.1997.tb02140.x, PMID: 9401419

[ref6] Berntson G. G. Stowell J. R. (1998). ECG artifacts and heart period variability: don’t miss a beat! Psychophysiology 35, 127–132. doi: 10.1111/1469-8986.3510127, PMID: 9499713

[ref7] Blevins C. A. Weathers F. W. Davis M. T. Witte T. K. Domino J. L. (2015). The posttraumatic stress disorder checklist for DSM-5 (PCL-5): development and initial psychometric evaluation. J. Trauma. Stress 28, 489–498. doi: 10.1002/jts.22059, PMID: 26606250

[ref8] Bradley R. Greene J. Russ E. Dutra L. Westen D. (2005). A multidimensional meta-analysis of psychotherapy for PTSD. Am. J. Psychiatry 162, 214–227. doi: 10.1176/appi.ajp.162.2.214, PMID: 15677582

[ref9] Brewin C. R. Andrews B. Valentine J. D. (2000). Meta-analysis of risk factors for posttraumatic stress disorder in trauma-exposed adults. J. Consult. Clin. Psychol. 68, 748–766. doi: 10.1037/0022-006X.68.5.748, PMID: 11068961

[ref10] Conoscenti L. M. Vine V. Papa A. Litz B. T. (2009). “Scanning for danger: readjustment to the noncombat environment,” in Living and Surviving in harm’s Way: A Psychological Treatment Handbook for Pre and Post-Deployment of Military Personnel. eds. Freeman S. M. Moore B. A. Freeman A. (New York, NY: Routledge), 126–145.

[ref11] Creaser J. Storr J. Karl A. (2021). Brain responses to a self-compassion induction in trauma survivors with and without PTSD. Frontiers in Psychology (in press). 10.3389/fpsyg.2022.765602 PMC8980710 35391975

[ref12] Dahm K. Meyer E. C. Neff K. Kimbrel N. A. Gulliver S. B. Morissette S. B. (2015). Mindfulness, self-compassion, posttraumatic stress disorder symptoms, and functional disability in U.S. Iraq and Afghanistan war veterans. J. Trauma. Stress 28, 460–464. doi: 10.1002/jts.22045, PMID: 26426991 PMC5032647

[ref13] Daneshvar S. Shafiei M. Basharpoor S. (2020). Group-based compassion-focused therapy on experiential avoidance, meaning-in-life, and sense of coherence in female survivors of intimate partner violence with PTSD: a randomized controlled trial. J. Interpers. Violence. doi: 10.1177/0886260520958660, PMID: [Epub ahead of print] 32933348

[ref14] Demers A. (2011). When veterans return: the role of community in reintegration. J. Loss Trauma 16, 160–179. doi: 10.1080/15325024.2010.519281

[ref15] Department of the Army (1984). *Field Manual (FM 21-75): Combat Skills of the Soldier*. Washington DC: Headquarters Department of the Army.

[ref16] DeVries C. A. Glasper E. R. Detillion C. E. (2003). Social modulation of stress responses. Physiol. Behav. 79, 399–407. doi: 10.1016/s0031-9384(03)00152-5, PMID: 12954434

[ref17] Dunn R. Brooks S. Rubin J. Greenberg N. (2015). Psychological impact of traumatic events. Occup. Health Work 12, 17–21.

[ref18] Ehlers A. Clark D. M. (2000). A cognitive model of posttraumatic stress disorder. Behav. Res. Ther. 38, 319–345. doi: 10.1016/s0005-7967(99)00123-0, PMID: 10761279

[ref19] Ehlers A. Clark D. M. (2009). Post-traumatic stress disorder: the development of effective psychological treatments. Nord. J. Psychiatry 62, 11–18. doi: 10.1080/08039480802315608, PMID: 18752113 PMC3059487

[ref20] Eisenberger N. I. Cole S. W. (2012). Social neuroscience and health: neurophysiological mechanisms linking social ties with physical health. Nat. Neurosci. 15, 669–674. doi: 10.1038/nn.3086, PMID: 22504347

[ref21] Faul F. Erdfelder E. Bucher A. Lang A. G. (2009). Statistical power analyses using G*Power 3.1: tests for correlation and regression analyses. Behav. Res. Methods 41, 1149–1160. doi: 10.3758/BRM.41.4.1149, PMID: 19897823

[ref22] Fear N. T. Jones M. Murphy D. Hull L. Iversen A. C. Coker B.. (2010). What are the consequences of deployment to Iraq and Afghanistan on the mental health of the UK armed forces? A cohort study. Lancet 375, 1783–1797. doi: 10.1016/S0140-6736(10)60672-1, PMID: 20471076

[ref23] Feiring C. Taska L. S. (2005). The persistence of shame following sexual abuse: a longitudinal look at risk and recovery. Child Maltreat. 10, 337–349. doi: 10.1177/1077559505276686, PMID: 16204736

[ref25] Freedman S. A. Gilad M. Ankri Y. Roziner I. Shalev A. Y. (2015). Social relationship satisfaction and PTSD: which is the chicken and which is the egg? Eur. J. Psychotraumatol. 6:28864. doi: 10.3402/ejpt.v6.28864, PMID: 26684986 PMC4696463

[ref26] Germain A. Neilsen T. (2003). Sleep pathophysiology in posttraumatic stress disorder and idiopathic nightmare sufferers. Biol. Psychiatry 54, 1092–1098. doi: 10.1016/s0006-3223(03)00071-4, PMID: 14625152

[ref27] Germer C. K. Neff K. D. (2013). Self-compassion in clinical practice. J. Clin. Psychol. 69, 856–867. doi: 10.1002/jclp.22021, PMID: 23775511

[ref28] Germer C. K. Neff K. D. (2015). “Cultivating self-compassion in trauma survivors,” in Mindfulness-Oriented Interventions for Trauma: Integrating Contemplative Practices. eds. Follette V. M. Briere J. Rozelle D. Hopper J. W. Rome D. I. (New York, NY, US: Guilford Press), 43–58.

[ref29] Gilbert P. (2009a). The Compassionate Mind. London: Constable Robinson.

[ref30] Gilbert P. (2010). An introduction to compassion focused therapy in cognitive behaviour therapy. J. Cogn. Psychother. 3, 97–112. doi: 10.1521/ijct.2010.3.2.97

[ref31] Gillath O. Noftle E. E. Stockdale G. D. (2009). Development and validation of a State Adult Attachment Measure (SAAM). J. Res. Pers. 43, 362–373. doi: 10.1016/j.jrp.2008.12.009

[ref32] Gross J. J. (1998). Antecedent and response-focused emotion regulation: divergent consequences for experience, expression, and physiology. J. Pers. Soc. Psychol. 74, 224–237. doi: 10.1037//0022-3514.74.1.224, PMID: 9457784

[ref33] Gross J. J. John O. P. (2003). Individual differences in two emotion regulation processes: implications for affect, relationships, and well-being. J. Pers. Soc. Psychol. 85, 348–362. doi: 10.1037/0022-3514.85.2.348, PMID: 12916575

[ref34] Gutner C. A. Pineles S. L. Griffin M. G. Bauer M. R. Weierich M. R. Resick P. A. (2010). Physiological predictors of posttraumatic stress disorder. J. Trauma. Stress 23, 775–784. doi: 10.1002/jts.20582, PMID: 21171139 PMC3336199

[ref36] Hiraoka R. Meyer E. C. Kimbrel N. A. DeBeer B. B. Gulliver S. B. Morissette S. B. (2015). Self-compassion as a prospective predictor of PTSD symptom severity among trauma-exposed U.S. Iraq and Afghanistan war veterans. J. Trauma. Stress 28, 127–133. doi: 10.1002/jts.21995, PMID: 25808565 PMC5032642

[ref37] Hogberg G. Pagani M. Sundin O. Soares J. Aberg-Wistedt A. Tarnell B.. (2008). Treatment of post-traumatic stress disorder with eye movement desensitization and reprocessing: outcome is stable in 35-month follow-up. Psychiatry Res. 159, 101–108. doi: 10.1016/j.psychres.2007.10.019, PMID: 18336919

[ref38] Hoge C. W. Castro C. A. Messer S. C. McGurk D. Cotting D. I. Koffman R. L. (2004). Combat duty in Iraq and Afghanistan, mental health problems and barriers to care. N. Engl. J. Med. 351, 13–22. doi: 10.1056/NEJMoa040603, PMID: 15229303

[ref39] Hotopf M. Hull L. Fear N. T. Browne T. Horn O. Iversen A.. (2003). The health of UK military personnel who deployed to the 2003 Iraq war: a cohort study. Lancet 367, 1731–1741. doi: 10.1016/S0140-6736(06)68662-5, PMID: 16731268

[ref40] Hutcherson C. A. Seppala E. M. Gross J. J. (2008). Loving-kindness meditation increases social connectedness. Emotion 8, 720–724. doi: 10.1037/a0013237, PMID: 18837623

[ref41] Kang H. K. Natelson B. H. Mahan C. M. Lee K. Y. Murphy F. M. (2003). Post-traumatic stress disorder and chronic fatigue syndrome-like illness in Gulf War veterans: A population-based survey of 30,000 veterans. Am. J. Epidemiol. 157, 141–148. doi: 10.1093/aje/kwf187, PMID: 12522021

[ref101] Kearney D. J. Malte C. A. McManus C. Martinez M. E. Felleman B. Simpson T. L. (2013). Loving-kindness meditation for posttraumatic stress disorder: a pilot study. J. Trauma. Stress 26, 426–434. doi: 10.1002/jts.21832, PMID: 23893519

[ref42] Kelley M. L. Bravo A. J. Davies R. L. Hamrick H. C. Vinci C. Redman J. C. (2019). Moral injury and suicidality among combat-wounded veterans: The moderating effects of social connectedness and self-compassion. Psychol. Trauma Theory Res. Pract. Policy 11, 621–629. doi: 10.1037/tra0000447, PMID: 30896225 PMC7224359

[ref43] Kimble M. O. Fleming K. Bennion K. A. (2013). Contributors to hypervigilance in a military and civilian sample. J. Interpers. Violence 28, 1672–1692. doi: 10.1177/0886260512468319, PMID: 23334188 PMC4157995

[ref44] King D. W. Taft C. King L. A. Hammond C. Stone E. R. (2006). Directionality of the association between social support and posttraumatic stress disorder: a longitudinal investigation. J. Appl. Soc. Psychol. 36, 2980–2992. doi: 10.1111/j.0021-9029.2006.00138.x

[ref46] Kirschner H. Kuyken W. Karl A. (2021). A biobehavioural approach to understand how mindfulness-based cognitive therapy reduces dispositional negative self-bias in recurrent depression. doi: 10.31234/osf.io/53trs [Epub ahead of print]

[ref47] Kirschner H. Kuyken W. Wright K. Roberts H. Brejcha C. Karl A. (2019). Soothing your heart and feeling connected: A new experimental paradigm to study the benefits of self-compassion. Clin. Psychol. Sci. 7, 545–565. doi: 10.1177/2167702618812438, PMID: 32655984 PMC7324152

[ref48] Kok B. C. Herrell R. K. Thomas J. L. Hoge C. W. (2012). Posttraumatic stress disorder associate with combat service in Iraq or Afghanistan: reconciling prevalence differences between studies. J. Nerv. Ment. Dis. 200, 444–450. doi: 10.1097/NMD.0b013e3182532312 22551799

[ref49] Kroenke K. Spitzer R. L. Williams J. B. (2001). The PHQ-9: validity of a brief depression severity measure. J. Gen. Intern. Med. 16, 606–613. doi: 10.1046/j.1525-1497.2001.016009606.x, PMID: 11556941 PMC1495268

[ref50] Kuyken W. Watkins E. Holden E. White K. Taylor R. S. Byford S.. (2010). How does mindfulness-based cognitive therapy work? Behav. Res. Ther. 48, 1105–1112. doi: 10.1016/j.brat.2010.08.003, PMID: 20810101

[ref51] Lazarus R. S. Alfert E. (1964). Short-circuiting of threat by experimentally altering cognitive appraisal. J. Abnorm. Soc. Psychol. 69, 195–205. doi: 10.1037/h0044635, PMID: 14213291

[ref52] Leaviss J. Uttley L. (2015). Psychotheraputic benefits of compassion-focused therapy: an early systematic review. Psychol. Med. 45, 927–945. doi: 10.1017/S0033291714002141, PMID: 25215860 PMC4413786

[ref53] Lee D. A. (2009). “Compassion-focused cognitive therapy for shame-based trauma memories and flashbacks in post-traumatic stress disorder,” in A Casebook of Cognitive Therapy for Traumatic Stress Reactions. ed. Grey N. (UK: Routledge), 230–245.

[ref54] Litz B. T. Stein N. Delaney E. Lebowitz L. Nash W. P. Silva C.. (2009). Moral injury and moral repair in war veterans: A preliminary model and intervention strategy. Clin. Psychol. Rev. 29, 695–706. doi: 10.1016/j.cpr.2009.07.003, PMID: 19683376

[ref55] Long P. Neff K. D. (2018). Self-compassion is associated with reduced self-presentation concerns and increased student communication behavior. Learn. Individ. Differ. 67, 223–231. doi: 10.1016/j.lindif.2018.09.003

[ref56] Lykken D. T. Rose R. Luther B. Maley M. (1966). Correcting psychophysiological measures for individual differences in range. Psychol. Bull. 66, 481–484. doi: 10.1037/h0023922, PMID: 5974620

[ref57] MacBeth A. Gumley A. (2012). Exploring compassion: A meta-analysis of the association between self-compassion and psychopathology. Clin. Psychol. Rev. 32, 545–552. doi: 10.1016/j.cpr.2012.06.003, PMID: 22796446

[ref58] Mantzios M. (2014). Exploring the relationship between worry and impulsivity in Military Recruits: the role of mindfulness and self-compassion as potential mediators. Stress. Health 30, 397–404. doi: 10.1002/smi.2617, PMID: 25476964

[ref59] McAllister L. Callaghan J. E. M. Fellin L. C. (2018). Masculinities and emotional expression in UK servicemen: ‘Big boys don’t cry’? J. Gend. Stud. 28, 257–270. doi: 10.1080/09589236.2018.1429898

[ref60] Meyer E. C. Szabo Y. Z. Frankfurt S. B. Kimbrel N. A. DeBeer B. B. Morissette S. B. (2019). Predictors of recovery from post-deployment posttraumatic stress disorder symptoms in war veterans: The contributions of psychological flexibility, mindfulness, and self-compassion. Behav. Res. Ther. 114, 7–14. doi: 10.1016/j.brat.2019.01.002, PMID: 30658166 PMC8441987

[ref61] Mintz J. Luborsky L. Christoph P. (1979). Measuring the outcomes of psychotherapy: findings of the Penn Psychotherapy Project. J. Consult. Clin. Psychol. 47, 319–334. doi: 10.1037/0022-006X.47.2.319, PMID: 469080

[ref62] Mounce L. T. A. Jones J. M. Jetten J. Haslam S. A. Williams W. H. (2013). Neurogenic and psychogenic acute Postconcussion symptoms can be identified After mild traumatic brain injury. J. Head Trauma Rehabil. 28, 397–405. doi: 10.1097/HTR.0b013e318252dd75, PMID: 22691962

[ref63] Neff K. (2003a). Development and validation of a scale to measure self-compassion. Self Identity 2, 223–250. doi: 10.1080/15298860390209035

[ref64] Neilson E. C. Singh S. R. Harper K. L. Teng E. J. (2020). Traditional masculinity ideology, posttraumatic stress disorder (PTSD) symptom severity, and treatment in service members and veterans: a systematic review. Psychol. Men Masculinities 21, 578–592. doi: 10.1037/men0000257

[ref65] Ozer E. J. Best S. R. Lipsey T. L. Weiss D. S. (2003). Predictors of posttraumatic stress disorder and symptoms in adults: a meta-analysis. Psychol. Bull. 129, 52–73. doi: 10.1037/0033-2909.129.1.52, PMID: 12555794

[ref66] Pearlman L. A. Curtois C. A. (2005). Clinical applications of the attachment framework: relational treatment of complex trauma. J. Trauma. Stress 18, 449–459. doi: 10.1002/jts.20052, PMID: 16281242

[ref100] Pietrzak R. H. Johnson D. C. Goldstein M. B. Malley J. C. Southwick S. M. (2009). Psychological resilience and postdeployment social support protect against traumatic stress and depressive symptoms in soldiers returning from operations enduring freedom and Iraqi freedom. Depression and Anxiety 26, 745–751. doi: 10.1002/da.20558, PMID: 19306303

[ref67] Pole N. (2007). The psychophysiology of posttraumatic stress disorder: a meta-analysis. Psychol. Bull. 133, 725–746. doi: 10.1037/0033-2909.133.5.725, PMID: 17723027

[ref68] Prescott M. R. (2012). The differences between war and civilian related traumatic events and the presentation of posttraumatic stress disorder and suicidal ideation in a sample of National Guard soldiers. Doctoral dissertation. University of Michigan.

[ref69] Rabon J. K. Hirsch J. K. Kaniuka A. R. Sirois F. Brooks B. D. Neff K. (2019). Self-compassion and suicide risk in veterans: when the going gets tough, do the tough benefit more from self-compassion? Mindfulness 10, 2544–2554. doi: 10.1007/s12671-019-01221-8

[ref70] Raes F. Pommier E. Neff K. D. Van Gucht D. (2011). Construction and factorial validation of a short form of the Self-Compassion Scale. Clin. Psychol. Psychother. 18, 250–255. doi: 10.1002/cpp.702, PMID: 21584907

[ref71] Reit R. (2009). The relationship between the Military’s masculine culture and service member’s help-seeking behaviours. Doctoral dissertation. Marquette University.

[ref72] Saraiya T. Lopex-Castor T. (2016). Ashamed and afraid: a scoping review of the role of shame in post-traumatic stress disorder (PTSD). J. Clin. Med. 5:94. doi: 10.3390/jcm5110094, PMID: 27809274 PMC5126791

[ref74] Solomon D. A. Keller M. B. Leon A. C. Mueller T. I. Lavori P. W. Shea T.. (2000). Multiple recurrences of major depressive disorder. Am. J. Psychiatr. 157, 229–233. doi: 10.1176/appi.ajp.157.2.229, PMID: 10671391

[ref75] Southwick S. M. Vythilingam M. Charney D. S. (2005). The psychobiology of depression and resilience to stress: implications for prevention and treatment. Annu. Rev. Clin. Psychol. 1, 255–291. doi: 10.1146/annurev.clinpsy.1.102803.143948, PMID: 17716089

[ref76] Steen M. P. Di Lemma L. Finnegan A. Wepa D. McGhee S. (2021). Self-compassion and veteran’s health: A scoping review. J. Veterans Stud. 7, 86–130. doi: 10.21061/jvs.v7i1.219

[ref78] Tabachnick B. G. Fidell L. S. (2007). Using Multivariate Statistics (5th Edn). Boston, MA: Allyn & Bacon/Pearson Education.

[ref79] Taft C. Kaloupek D. Schumm J. Marshall A. Panuzio J. King D.. (2007). Posttraumatic stress disorder symptoms, physiological reactivity, alcohol problems, and aggression among military veterans. J. Abnorm. Psychol. 116, 498–507. doi: 10.1037/0021-843X.116.3.498, PMID: 17696706

[ref80] Thompson B. Waltz J. (2008). Self-compassion and PTSD symptom severity. J. Trauma. Stress. 21, 556–558. doi: 10.1002/jts.20374, PMID: 19107727

[ref81] Tick E. (2005). War and the Soul: Health our nation’s Veterans from Post-Traumatic Stress Disorder. Wheaton, IL: Quest Books.

[ref82] Weathers F. W. Litz B. T. Keane T. M. Palmieri P. A. Marx B. P. Schnurr P. P. (2013). The PTSD Checklist for DSM-5 (PCL-5) – Standard [Measurement instrument]. Available at: https://www.ptsd.va.gov/

[ref83] Wessely S. (2006). Twentieth-century theories on combat motivation and breakdown. J. Contemp. Hist. 41, 268–286. doi: 10.1177/0022009406062067

[ref84] Williams W. H. Cordan G. Mewse A. J. Tonks J. Burgess C. N. W. (2010). Self-reported traumatic brain injury in male young offenders: a risk factor for re-offending, poor mental health and violence? Neuropsychol. Rehabil. 20, 801–812. doi: 10.1080/09602011.2010.519613, PMID: 21069616

[ref85] Williams R. H. Zimmerman D. W. Rich J. M. Steed J. L. (1984). Empirical estimates of the validity of four measures of change. Percept. Mot. Skills 58, 891–896. doi: 10.2466/pms.1984.58.3.891

